# Biosynthesis and the Transcriptional Regulation of Terpenoids in Tea Plants (*Camellia sinensis*)

**DOI:** 10.3390/ijms24086937

**Published:** 2023-04-08

**Authors:** Junchi Wei, Yun Yang, Ye Peng, Shaoying Wang, Jing Zhang, Xiaobo Liu, Jianjun Liu, Beibei Wen, Meifeng Li

**Affiliations:** College of Tea Science, Guizhou University, Guiyang 550025, China

**Keywords:** terpenoid biosynthesis, MEP and MVA pathways, biotic and abiotic stress factors, transcriptional regulation, *Camellia sinensis*

## Abstract

Terpenes, especially volatile terpenes, are important components of tea aroma due to their unique scents. They are also widely used in the cosmetic and medical industries. In addition, terpene emission can be induced by herbivory, wounding, light, low temperature, and other stress conditions, leading to plant defense responses and plant–plant interactions. The transcriptional levels of important core genes (including *HMGR*, *DXS*, and *TPS*) involved in terpenoid biosynthesis are up- or downregulated by the MYB, MYC, NAC, ERF, WRKY, and bHLH transcription factors. These regulators can bind to corresponding cis-elements in the promoter regions of the corresponding genes, and some of them interact with other transcription factors to form a complex. Recently, several key terpene synthesis genes and important transcription factors involved in terpene biosynthesis have been isolated and functionally identified from tea plants. In this work, we focus on the research progress on the transcriptional regulation of terpenes in tea plants (*Camellia sinensis*) and thoroughly detail the biosynthesis of terpene compounds, the terpene biosynthesis-related genes, the transcription factors involved in terpene biosynthesis, and their importance. Furthermore, we review the potential strategies used in studying the specific transcriptional regulation functions of candidate transcription factors that have been discriminated to date.

## 1. Introduction

Terpenoids are the most abundant secondary metabolites of plants with diverse structures and aromatic scents [1,2,3]. The chemical structures of terpenoids are extremely variable yet share the common feature of biosynthesis, and the number of five-carbon isoprene units in the skeleton structure is the key for the classification of terpenoids [4,5,6,7]. Isoprene (C_5_), monoterpenes (C_10_), sesquiterpenes (C_15_), diterpenoids (C_20_), sesterterpenes (C_25_), triterpenes (C_30_), and tetraterpenes (C_40_) are important terpenoid substances [8,9,10]. It is well known that terpenoids play an important role in plant life through direct and indirect plant defense, pollinator attraction, and different interactions between plants and their environment [11,12,13,14]. Monoterpenes (linalool, limonene, myrcene, and trans-β-ocimene) and some sesquiterpenes (farnesene, nerolidol, and caryophyllene) are common constituents of floral scents [4,15,16,17]. In addition, these terpenoid substances might serve as important signals mediating interactions between plants and herbivores, and they have potential applications in the pharmaceutical, food, and cosmetic industries [6,18,19,20,21].

The mevalonic acid (MVA) and methylerythritol phosphate (MEP) pathways are two independent, compartmentally separated pathways for terpenoid formation [22,23,24,25]. The MEP pathway is mainly responsible for the biosynthesis of mono- and diterpenes, while the MVA pathway mainly produces sesquiterpenes, sterols, and triterpenes in plants [1,26]. Linear prenyl diphosphates are the basic building blocks for C_5_ and can be produced from the condensation of IPP and DMAPP by prenyltransferases [1]. The precursor of monoterpenes, geranyl pyrophosphate (GPP), is formed from one DMAPP and one IPP molecule by GPP synthase (GPPS) in plastids [27]. Moreover, the precursor of sesquiterpenes, farnesyl diphosphate (FPP), is synthesized from two IPP molecules and one DMAPP molecule by farnesyl diphosphate (FPP) synthase in the cytosol [28]. The tremendous diversity of volatile terpenoids in plants is mainly ascribed to the ample catalytic versatility of terpene synthases (TPSs), many of which have the distinctive ability to synthesize multiple products from prenyl diphosphate precursors, GPP, FPP, and geranylgeranyl diphosphate (GGPP) [29,30]. Since the isolation and purification of *S*-linalool synthase (LIS) from the flower fragrance in *Clarkia breweri* by Pichersky [31], some *TPS* genes related to floral scent biosynthesis have been gradually reported, such as in citrus (*Citrus sinensis*) [32], carrot (*Daucus carota*) [33], tea (*Camellia sinensis*) [34], and sweet pea (*Lathyrus odoratus*) [35]. The *TPS* family genes constitute a medium-sized to large family. There are approximately 20–150 functional members in the genomes of almost all plant species [36], and a total of 80 *TPS-like* genes have been identified in tea plants to date [34].

The biosynthesis of terpenoids is reported to be regulated by a variety of transcription factors. For example, MYB and MYC were found to work cooperatively in controlling the expression of terpene synthase genes. In the *myb21* mutant of *Arabidopsis*, it was observed that the emission of sesquiterpenes catalyzed by *AtTPS11* and *AtTPS21* was decreased, but whether AtMYB21 could regulate *AtTPS11* and *AtTPS21* directly or not was unclear. Moreover, AtMYB21 and AtMYC2 might form the MYB–bHLH complex to regulate the biosynthesis of sesquiterpenes [37]. Recently, the highly transactive MYB transcription factor in *Freesia hybrida*, FhMYB21L2, was demonstrated to activate the expression of *FhTPS1* via binding to the MYBCORE motif (CAACCG) in its promoter region. On the other hand, FhMYC2 exhibited a negative effect on the expression of *FhTPS1* by trapping the FhMYB21L2, which might be the proposed regulatory mechanism for the dynamic change of linalool emission at different developmental stages of the *Freesia* flowers. In addition, the MYC2 transcription factor was found to interact with RERJ1 (a bHLH transcription factor) [38] or DELLA proteins to regulate the expression of sesquiterpene synthase genes [39]. Other transcription factors, such as NAC, EIN3-like [40], HY5 (bZIP transcription factor) [41], bHLH [42], and scarecrow-like (SCL) [43], have also been found to participate in regulating the expression of *TPSs*.

Tea plants are evergreen perennial plants that originated in southwest China [44]. Although the *TPS* gene family has been identified and classified in tea plants [34], the functions of most *CsTPSs* in tea plants remain unknown [45]. Studies based on the integration of metabolomics and transcriptomics have suggested that the MYB, MYC, bHLH, NAC, ERF, and WRKY transcription factors may regulate tea aroma through the transcriptional regulation of the MVA pathway- and MEP pathway-related genes, thereby causing changes in terpenoid volatiles in tea leaves in different seasons [46,47,48]. This article discusses the recent advances in understanding the functions and molecular mechanisms of terpenoid biosynthesis and the transcriptional regulation of terpenoid production by different types of transcription factors in tea plants.

## 2. Terpenoid Compounds in Tea Plants

**Figure 1:**
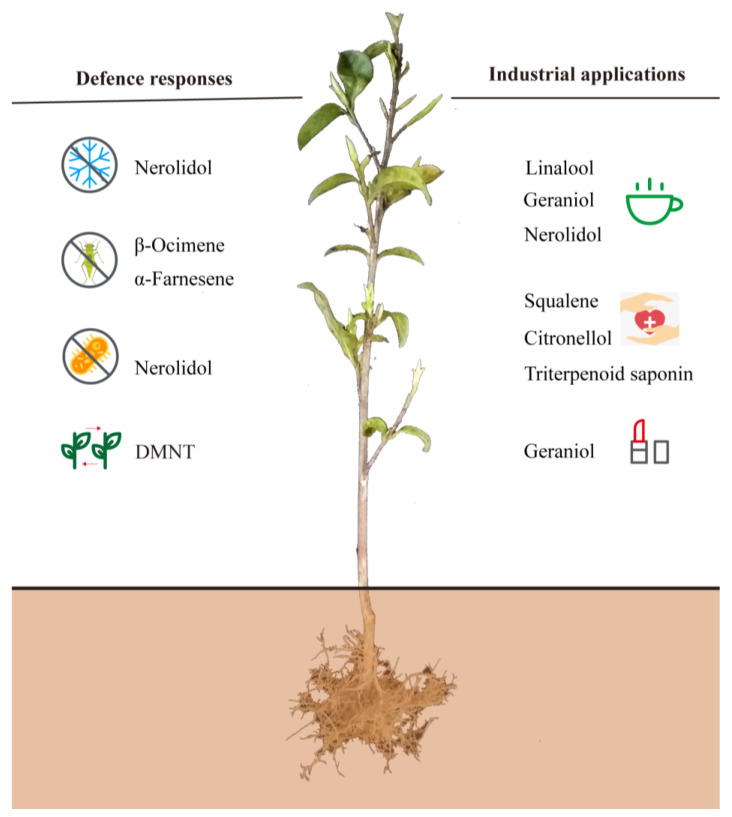
Important functions of main terpenoids in tea plants. DMNT, (3*E*)-4,8-Dimethyl-1,3,7-nonatriene.

Terpenes, or terpenoids, include more than 50,000–80,000 compounds with different structures, which are important resources for building essential isoprenoid compounds, such as sterols, brassinosteroids, cytokinins, quinones, chlorophyll, tocopherols, carotenoids, abscisic acid (ABA), and gibberellins [49,50]. Although some terpenes are primarily responsible for floral scents, including their aromas and physiological effects, most members of the terpenes are defensive toxins and herbivore deterrents [51]. This article summarizes the main functions of terpenoids in tea plants according to the research published in recent years (Figure 1). From the perspective of defense responses, it is well known that volatile terpenes play essential roles in communication between plants and in the communication between plants and other organisms, thus improving plant fitness (for example, through preventing herbivory and improving the success rate of pollination) [52]. Because of their special floral scents, some terpenes are widely used in industrial applications, such as in the pharmaceutical and cosmetic industries [6,18,53].

### 2.1. Terpenoids in Tea Aroma

Tea is the most popular beverage worldwide apart from water due to its sensory qualities and health benefits [54]. As an important component of tea quality, aroma has received great attention in tea production [55]. Terpenoids, along with lipids, carotenoids, phenylpropanoids, and their glycoside derivatives, constitute the main volatile organic compounds (VOCs) in tea aroma [56,57]. Although terpenoid volatiles have a relatively low detection threshold, they have been reported as key aroma compounds for the sensory quality of tea [46]. There are more than 100 types of terpenoid tea aroma substances, most of which are volatile monoterpenoids (such as geraniol and linalool) and sesquiterpenes (such as nerolidol) [34,58]. Studies have shown that volatile terpenes dominate the aroma (more than 60%) in tea products [59,60].

Generally speaking, β-myrcene, α-phellandrene, (*Z*)-β-ocimene, (*Z*)-furan linalool oxide, (*E*)-furan linalool oxide, γ-terpinene, DMNT, cosmene, (*E*)-pyranoid linalool oxide, linalool oxide pyranoside, linalyl formate, α-cubebene, γ-elemene, humulene, (*E*)-β-famesene, (*Z*, *E*)-α-farnesene, α-muurolene, α-farnesene, T-muurolol, α-cadinol, α-terpineol, geraniol, β-caryophyllen, δ-cadinene, (*E*)-nerolidol, linalool, D-limonene, and β-pinene are reported to be important terpene compounds in tea aroma [48,59,61]. Among them, linalool and its furan oxides, (*Z*)-furan linalool oxide and (*E*)-furan linalool oxide, are important components of sweet and floral aromas, while (*E*)-pyranoid linalool oxide and linalool oxide pyranoside have earthy aromas [62]. Geraniol and linalool oxides present pleasant floral scents and may be the principal contributors to the floral and green odor of Longjing tea [63,64,65]. The compound (*E*)-nerolidol has floral green, citrus woody, and waxy odors [59], which are among the characteristic aromas of oolong tea [66]. The content of these terpenes varies among different tea plant species and might affect the suitability of tea cultivars, indicating that they may play a major role in the formation of floral fragrance [59].

Although many aroma compounds are found in tea, only a small number of components whose concentration exceeds their odor threshold contribute to the aroma characteristics of tea. Different fragrances have different threshold concentrations because the detection limits of the human olfactory system are different. In some cases, the threshold concentration between the two different aroma components differs by a thousand times [67]. Linalool, geraniol, and nerolidol are potent odorants of tea, with flavor dilution (FD) factors of 64, 64, and 32, respectively. These compounds impart tea products with a creamy, rose-like floral odor [68,69]. Three terpenoids, β-myrcene, D-limonene, and (*E*)-α-farnesene, are considered aroma-active compounds and have been detected in Rougui Wuyi rock tea, especially β-myrcene, which has a spicy aroma that might contribute to herbal or woody odors [69]. In white tea, volatile terpenes, including β-myrcene, linalool, and geraniol, are the key potent odorants, and geraniol and linalool mainly contribute a strong floral aroma when tea leaves are withered under sunlight [70]. In addition, some terpene substances that do not contribute to human sensory perception are also involved in sensory formation by indirectly affecting the formation of other aroma components or in response to adverse processes [59].

### 2.2. Responses of Terpenoids to Stress

Plants synthesize and release many types of VOCs for reproduction, defense, and communication between plants [71,72,73]. The formation of volatile terpenoids can be influenced by various stresses, including biotic stress (such as tea green leafhopper herbivory) and abiotic stress (such as light, temperature, and wounding) during the tea plant’s growth process and the manufacture of tea products [67]. At present, little is known about the specific mechanism through which plants sense volatiles sent by other plants [52]. Some modern strategies, such as *E. coli*, yeast expression systems, and plant transgenic systems, can be applied to the investigation of the relationships between characteristic aroma compounds and plants [67].

The direct defenses of plants include physical structures (trichomes and thorns) and the accumulation of chemical or biochemical compounds induced by herbivore feeding, most of which exhibit antibiotic activities or toxicity [74]. Some of the sesquiterpenoids provide direct protection through the formation of phytoalexins, which are produced as part of the plant’s defense system [75]. Volatiles emitted from damaged vegetative tissues after herbivore feeding have been reported to exhibit the function of protecting plants by deterring herbivores and by attracting the enemies of herbivores [5]. It has been established that herbivory-induced volatile terpenoids may serve as both indirect and direct defenses [76,77]. After egg deposition or feeding by herbivorous arthropods, plants can be induced to emit volatile terpenoids [78,79], such as geraniol, farnesene, ocimenes, linalool, and nerolidol [80]. These volatile blends can be exploited by natural enemies of the herbivores to locate infested plants (predators and parasitoids), and the release of these terpenoids is considered to be an important mechanism for plants to indirectly defend themselves [81]. Herbivore-specific compounds from the oral secretions of feeding insects might be the underlying cause of a rapid change in the green leaf volatiles emitted from plants [79]. DMNT, a common volatile released in response to herbivore attacks and a floral odor constituent, was identified as an effective compound used by herbivorous insects to find their host plants for feeding and egg deposition [82]. After being attacked by the geometrid *Ectropis obliqua*, the emission of DMNT was significantly increased, and it seems to have interacted with jasmonic acid (JA) to promote the resistance of neighboring intact plants to herbivorous insects [83].

In addition to biotic stresses, abiotic stresses have also been found to increase the concentration of most volatile terpenes significantly [84]. Recent studies have demonstrated that light can activate the formation of plant volatiles, while the metabolite levels of tea leaves require a relatively long time to respond to light treatment. In contrast to natural light or dark treatment, blue light (470 nm) and red light (660 nm) significantly increased most endogenous volatile terpenes via significantly upregulating the expression levels of key genes involved in volatile terpene formation [84]. However, green light irradiation could markedly damage the aroma and taste of the tea, leading to a strong greenish flavor and an astringent taste, probably because green light irradiation decreased the contents of chemical compounds in black tea [85]. Furthermore, UV-B treatment application on one-year-old potted plants of *C. sinensis* cv. “Longjing-43” differentially altered the metabolism of terpenoids with significant effects at 8 h of treatment, demonstrating the strong potential for UV-B application in flavor improvement in tea [86]. During the processing of tea, the combination of a low-temperature application and wounding damage has been demonstrated to improve tea aroma [87]. For example, (*E*)-nerolidol can be induced during the turn over stage with the mechanical damage at a relatively low temperature, which is not detected in intact tea plants [88]. In addition, drought and wounding caused by long-term withering (36–48 h) significantly induced the upregulated expression of monoterpenes, such as linalool and geraniol, while floral and fruit flavor compounds ((*E*)-nerolidol and cis-jasmone) showed a significant decrease in content concomitantly [58].

### 2.3. Potential Uses of Terpenoids in Industrial Applications

In addition to distinguishing the importance of some terpenes in the tea aroma quality due to their unique floral scents as discussed in Section 2.1. of the present work, terpenoids in tea plants have been widely applied in the medical and cosmetic industries, including squalene, citronellol, triterpenoid saponin, and geraniol [89,90,91]. Squalene (C_30_H_50_) is an intermediate hydrocarbon in the biosynthesis of terpenes that was first found in shark liver oil. Since its discovery, squalene has been widely used in biological applications due to its beneficial properties, including its anticarcinogenic, antioxidant, and skin-hydrating properties, among others [92,93,94]. Due to the health properties of squalene and its rising demand in industrial uses, alternative sources from plants or microorganisms need to be identified given the recent restrictions on harvesting sharks [93,95,96]. The essential oils in medicinal plants have profound applications in treating central nervous system disorders and diseases of inflammatory etiology. Some of the medicinal plants are rich in secondary metabolites with antihyperalgesic activity, such as citronellal with antihyperalgesic activity [97]. Two enantiomers of citronellol, namely (*R*)-(+)-β-citronellol and (*S*)-(−)-β-citronellol, are distributed in many medicinal and aromatic plants [98]. Triterpenoid saponins are recognized for their medicinal benefits, such as acylated oleanane-type triterpene oligoglycosides and florathea saponins A–C in tea plants [99,100]. Oleanane-type triterpenoid saponins exhibit antiproliferative activity against digestive carcinoma human cell lines, indicating that triterpenoid saponins may be a valuable tool to improve the efficacy of cytostatics [101,102]. Geraniol is known as an important ingredient in many highly valued essential oils for its rose-like aroma and is widely applied in cosmetic products [103]. Fragrance is an integral character of cosmetics, and some essential oils of citrus, lavender, eucalyptus, and tea tree are often used as fragrances in the cosmetic industry to stimulate the interest among consumers [104,105].

## 3. Biosynthesis Pathways of Terpenoids

**Figure 2:**
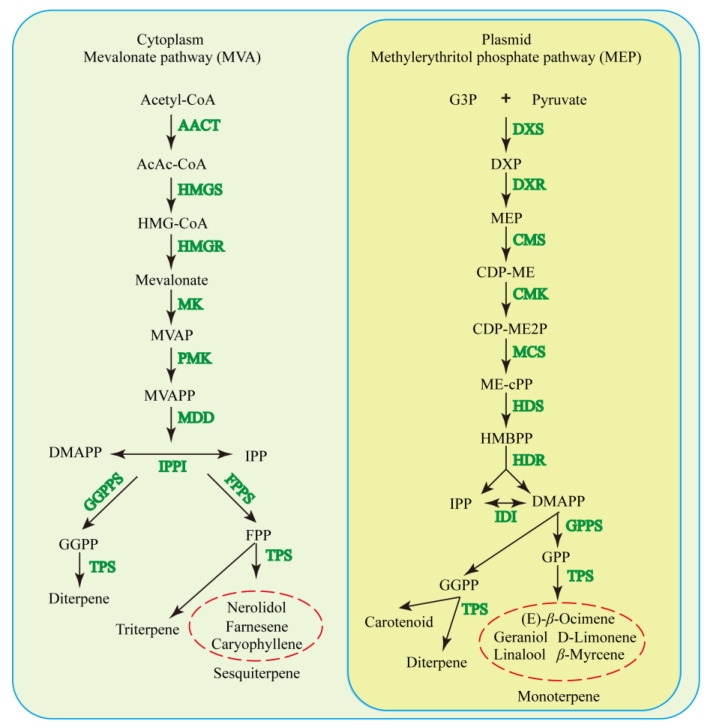
Terpenoid biosynthesis pathways in plants. MVA, mevalonic acid; AACT, acetyl-CoA acetyltransferase; HMG, 3-hy-droxy-3-methylglutaryl-CoA; HMGS, hydroxy methylglutaryl-CoA synthase; HMGR, hydroxy methylglutaryl-CoA reductase; MK, mevalonate kinase; MVAP, mevalonate-5-phosphate; PMK, phosphomevalonate kinase; MVAPP, mevalonate-5-diphosphate; MDD, mevalonate-5-diphosphate decarboxylase; IPP, isopentenyl diphosphate; IPPI, isopentenyl diphosphate isomerase; DMAPP, dimethylallyl pyrophosphate; GGPPS, GGPP synthase; FPPS, farnesyl pyrophosphate synthase; FPP, farnesyl pyrophosphate; GGPP, geranylgeranyl pyrophosphate; TPS, terpene synthase/cyclase; MEP, methylerythritol phosphate; DXS, 1-deoxy-D-xylulose-5-phosphate synthase; DXP, 1-deoxy-D-xylulose-5-phosphate; DXR, 1-deoxy-D-xylulose-5-phosphate reductoisomerase; CMS, CDP-ME synthase; CDP-ME, 4-diphosphocytidyl-2-C-methyl-D-erythritol; CMK, 4-diphosphocytidyl-2-C-methyl-D-erythritol kinase; CDP-ME2P, 4-(cytidine-5′ -diphospho)-2-C-methyl-D-erythritol-2-phosphate; MCS, 2-C-methyl-D-erythritol-2,4-cyclodiphosphate synthase; ME-cPP, 2-C-methyl-d-erythritol 2, 4-cyclodiphosphate; HDS, 4-hydroxy-3-methylbut-2-enyl diphosphate synthase; HMBPP, 4-hydroxy-3-methylbut-2-enyl diphosphate; HDR, 4-hydroxy-3-methylbut-2-enyl diphosphate reductase; DMAPP, dimethylallyl diphosphate; IDI, IPP/DMAPP isomerase; GPPS, GPP synthase; and GPP, geranyl pyrophosphate.

Terpenoids contain the basic building block units for IPP and its isomer DMAPP, from two relatively separate pathways, namely the MEP and MVA pathways [27], in plastids and the cytosol, respectively (Figure 2). The MEP pathway is mainly responsible for the biosynthesis of mono- and diterpenes (~53 and ~1% of total floral terpenoids, respectively), and the MVA pathway is mainly responsible for the biosynthesis of sesquiterpenes (~28% of total floral terpenoids) [1,106]. Although these isoprenoid biosynthetic pathways are separated, they are connected by metabolic “cross-talk”, which is mediated by unrecognized transporters [1]. The MEP pathway begins with the condensation of D-glyceraldehyde 3-phosphate and pyruvate and involves seven enzymatic reactions, while the MVA pathway starts from the stepwise condensation of three molecules of acetyl-CoA and consists of six enzymatic reactions [107]. Then, two IPP molecules and one DMAPP molecule are synthesized to form FPP by FPP synthase in the cytosol, and one DMAPP with one IPP molecule in the plastids results in the formation of GPP, the precursor of monoterpenes, and is catalyzed by the GPPS [107]. TPS is responsible for the final steps in terpenoid biosynthesis (Figure 2) through catalyzing complex carbocation-driven cyclization, rearrangement, and elimination reactions [108]. Increasing evidence has shown that the TPS family has profound plastic functions in variable family sizes and evolving new enzymes with new functions [108,109,110]. The TPS family is classified into class I and class II TPSs [111]. Each group of TPSs has specific motifs that enable their distinctive functions, such as class I TPSs with the DDxx (D, E) motif and metal-binding NSE/DTE motif and class II TPSs with the DxDD motif [111,112,113].

It is generally believed that both class I and class II TPSs are responsible for the formation of hemiterpenes, monoterpenes, sesquiterpenes, diterpenes, sesterterpenes, and terpenes, while triterpenes and tetraterpenes are mainly synthesized by class II TPSs [114]. To date, few class I TPSs were also charactered to catalyze the formation of large-terpene, such as C_25_, C_30,_ and sesquarterpenes (C_35_) terpenes [114,115,116]. Isoprene (2-methyl-1,3-butadiene) is the most common form of hemiterpene and has the smallest and simplest C_5_ basic building block [117]. Isoprene emissions from plants play important roles in plant defenses against biotic and abiotic stresses, and they can also improve photosynthetic performance at high temperatures [118,119,120]. Monoterpenes are essential substances in the aromatic oil, cosmetic, perfume, food, and pharmaceutical industries for their unique odors [121,122,123]. While monoterpene synthases (mTPSs) are considered to catalyze GPP for monoterpenes, the GPP transporter has not yet been discovered [111]. The sesquiterpenes are constituents of floral scents and have health-promoting properties (such as antioxidant, anti-inflammatory, and anticancer properties), which might be mainly induced by ambient temperatures [124,125,126,127,128]. Sesquiterpenes are mainly synthesized in the cytosol through the MVA pathway with FPP as a substrate. In addition, the formation of small amounts of sesquiterpenes has been detected in plasmids [129,130]. Diterpene biosynthesis is well known to be initiated in plastids from GGPP in the MEP pathway [131,132,133]. Diterpene synthases (diTPSs) can be characterized as class I diTPSs and class II diTPSs, with the functions of forming additional rings, double bonds, or hydroxyl groups [134,135]. In addition, diTPS and cytochrome P450 monooxygenase (P450) enzymes have been found to work in combination to produce novel diterpene compounds [136,137,138]. Taxol, a famous diterpenoid secondary metabolite, is considered one of the most effective anticancer agents originally extracted from the bark of *Taxus brevifolia* [139]. In the taxol biosynthetic pathway, taxadiene synthase (TS), taxadiene-5α-hydroxy-lase (T5αOH), and specialized *Taxus* BAHD family acyltransferases (ACTs) are responsible for the cyclization of taxadiene and the modification of the taxane skeleton [140]. In addition, sclareol (labdane diterpenoid) is a natural starting material for ambrox and related ambroxide synthesis, because of its delicate odor and fixative properties [141]. In clary sage (*Salvia sclarea*), the main plant species for sclareol production, functional modification of labdane and labdane-related diterpenoids mainly involved the addition of hydroxy groups, which can be catalyzed by diTPSs and P450s [141,142]. Sesterterpenes consist of relatively rare terpenoid substances that are formed by geranylfarnesyl diphosphate synthase (GFPPS) using GFPP as the precursor in the plastid MEP pathway [143,144,145]. Triterpenes are C_30_ compounds derived from two molecules of FPP to generate the squalene catalyzed by squalene synthase (SQS) via the MVA pathway in the cytoplasm [26,146,147]. Although triterpenoids are not necessary for plant growth and development, the substances in this group have a wide range of biological activities and widespread commercial applications [148,149,150,151]. Tetraterpenes are derived from the phytoene condensation of isopentenyl diphosphate (IDP) and dimethylallyl diphosphate (DMADP) by phytoene synthase (PYS) [152]. Carotenoids are the most representative tetraterpenes and are famous natural functional pigments and photoprotectors that have demonstrated efficiency in preventing human health disorders [153,154,155,156].

## 4. Transcriptional Regulation of Volatile Terpenoids

### 4.1. Terpenoid Biosynthesis-Related Genes

To date, the terpene biosynthesis pathways (MEP and MVA) and many genes (such as *HMGR*, *DXS*, and *TPS*) related to terpenoid biosynthesis in tea plants have been extensively studied, and it has been demonstrated that TPSs of different classes catalyze the rate-limiting step of converting terpenoid precursors into monoterpenes, sesquiterpenes, and diterpenes [46,54]. The plant *TPS* gene family can be classified into the following subgroups: a (TPS-a, monofunctional class I sesqui- and di-TPSs), b (monofunctional class I mono-TPSs), c (TPS-c, monofunctional class II diTPSs), d (TPS-d, gymnosperm-specific class I mono-, sesqui-, di-TPSs, and bifunctional class II/I diTPSs), g (TPS-g, monofunctional class I mono-, sesqui-, and di-TPSs), e/f (TPS-e/f, monofunctional class I diTPSs), and h (TPS-h, Selaginella-specific bifunctional class II/I diterpene synthases (diTPSs)) [108]. Among them, TPS-a, TPS-b, and TPS-g are angiosperm-specific subgroups, while TPS-d is a gymnosperm-specific subgroup [36,108]. Few plants contain all the TPS subfamilies. Usually, the TPS family of each plant contains two or more subfamilies. A recent study identified 80 *TPS*-*like* genes in the *C. sinensis* cv. ‘Shuchazao’ (SCZ) genome, including TPS-a, TPS-b, TPS-c, TPS-g and TPS-e/f subgroups [34]. Interestingly, most sesquiterpene synthetized *CsTPSs* gained high transcriptional levels in flowers and leaves, while limited monoterpene synthase genes maintained substantial transcript levels in many tested organs. It was noted that the functions of the most-recognized *CsTPSs* remained unclear due to the limitation of sequence precision, indicating that more strategies need to be developed to obtain the full sequences of these genes for their functional validation in vitro and in plants. However, through the comprehensive comparison of several different tea plant genomes, it was found that the *CsTPS* family varied among different cultivars. For example, *CsTPS08* is only annotated in the genome of *C. sinensis* cv. ‘Huangdan’ (HD) and *C. sinensis* cv. ‘Tieguanyin’ (TGY) of the oolong tea species with higher terpene aroma, but not completely annotated in some tea species suitable for green tea processing [45].

Linalool is one of the most abundant and scent-determining constituents, including two isomers (*R*)-linalool and (*S*)-linalool, existing in plants [157,158,159]. Notably, (*R*)-linalool has been found to have a woodier and lavender-like aroma, while (*S*)-linalool has a sweet, floral, and petitgrain-like smell [158]. Linalool synthase (LIS) in tea plants specifically catalyzes the formation of linalool [160]. The transcript levels of *CsLISs* (*CsLIS1*, KF006849; *CsLIS2*, KR873396) were increased in tea leaves under single-wounding treatment and continuous-wounding treatment compared to that in fresh tea leaves, indicating that mechanical damage may promote the release of linalool from tea leaves [61]. Similarly, wounding stress during the turn over process was presumed to be the main factor to activate some key genes involved in the formation of volatiles, such as the linalool synthase gene *CsTPS2* (KR873395) [66]. *CsTPS42* (CSS0000049), a bifunctional enzyme defined as CsLIS/NES (NES, nerolidol synthase), can generate linalool with GPP as a substrate, and the upregulated expression levels of *CsTPS42* might lead to the corresponding release of linalool [48]. In addition, *CsTPS2* (KR873395) was also recognized as a linalool synthase gene that was significantly upregulated during the ‘withering’ step of oolong tea manufacture [66]. In conclusion, these previous studies indicate that the fact that the linalool synthase gene in tea plants might be proposed as a wounding stress-response gene. However, few studies have focused on the specific linalool stereoisomer-producing genes in tea plants. Zhou et al. [160] cloned two (*R*)-linalool synthase candidate genes, *CsRLIS* (MT178265) and *CsTPS* (XM_028210969), which specifically catalyzed the formation of (*R*)-linalool and caused the accumulation of internal (*R*)-linalool during oolong tea manufacture. Moreover, the relative expression levels of (*S*)-linalool synthase and (*R*)-linalool synthase genes might cause the dynamic levels of the proportions between two isomers of linalool among different *C. sinensis* cultivars.

The genes responsible for the biosynthesis of other essential terpene substances, such as nerolidol, α-farnesene, and β-ocimene, have also been studied in tea plants [46,48,60]. Nerolidol, a sesquiterpenoid alcohol, is the most effective allelopathic compound showing effective applications in agricultural practices [161,162,163,164]. In terms of health-promoting properties and floral scents, nerolidol is also known for its pharmaceutical and medicinal values [165,166,167]. NES is a key enzyme responsible for nerolidol biosynthesis, and some bifunctional linalool/nerolidol synthases severed by TPS can also produce nerolidol [46,165,168,169]. *CsTPS4* (KY033151), an *NES* gene, is significantly downregulated along with the withering degrees of white tea [60]. Based on the transcriptome analysis of the green tea spreading process, it was found that the expression level of *CsTPS35* (CSS0012706) was generally increased in different tea plant varieties, which was indicated to help produce (*E*)-nerolidol from FPP [48].The farnesyl pyrophosphate synthase (*FPS*) gene is a key enzyme gene in the terpenoid metabolism pathway that is crucial to the formation of tea quality and flavor [46]. The expression of *CsFPS* increases with the aggravation of drought, and the expression of *CsFPS* is upregulated under light conditions during the withering process of tea [170]. Ocimene (3,7-dimethyl-1,3,6-octatriene), a ubiquitous floral volatile compound in plants, can be emitted from flowers or vegetative tissues and can be used to attract pollinators or as an antiaphrodisiac pheromone in plant defense [171,172,173]. The FPKM-based gene expression profile of *CsOCS2* (OCS, β-ocimene synthase, and TEA004606.1) showed a high correlation not only with the accumulation of (*E*)-β-ocimene, but also with the other three monoterpenes (geraniol, β-myrcene, and D-limonene) and one sesquiterpene ((*E*)-β-fanesene) [46]. A new *CsOCS* gene (MN135992) that shared a low similarity to the previously characterized tea ocimene synthase genes (*CsOCS1*, TEA031457.1; *CsOCS2*, TEA004606.1) was isolated in ‘TGY’ tea plants [174]. The in vitro enzymatic reaction experiment indicted that CsOCS protein was a key enzyme responsible for a large amount of (*E*)-β-ocimene and a small amount of (*Z*)-β-ocimene using GPP as the substrate [174]. In addition, a plastid-located β-ocimene synthase gene *CsBOS1* (TRINITY_DN105425_c1_g2), was determined to be involved in the synthesis of β-ocimene in tea plants, which is especially sensitive to light treatment and the attack of tea geometrids [175]. Intriguingly, a *CsAFS* gene (GFMV01032657) was found that converted GPP to β-ocimene in vitro, revealing the bifunctional enzyme activity that is common in the *TPS* gene family [176]. Meanwhile, the diterpenoid-related genes in tea plants have recently been reported, including one CPS (*ent*-copalyl diphosphate synthase, CPS) and two highly similar KSs (*ent*-kaurene synthase, KS) [142]. In fast-growing tissues, such as tender stems and roots, the relative expression levels of *CsCPS* (MN961684) and *CsKSs* (MN961685; MN961686) exhibited highly coordinated patterns [142]. However, it seems that the 1% differential amino acids between CsKS1 and CsKS2 led to their functional divergence, according to the functional characterization experimental results [142]. In conclusion, the identification and definition of these structural genes have provided extended profiles for future transcriptional investigations.

### 4.2. Regulation of Transcription Factors Affects Terpenoid Biosynthesis

Many transcription factors have been confirmed to be involved in the regulation of plant terpenoids (Table 1). Among them, transcription factors that are involved in the secondary metabolism of plants (ERF and MYB) have been studied extensively. In maize (*Zea mays*), TPS10 mainly forms (*E*)-α- bergamotene and (*E*)-β-farnesene in leaves damaged by lepidopteran larvae. These compounds are highly attractive to the natural enemies of the herbivores [177]. EREB58, an AP2/ERF family transcription factor, was found to be a positive regulator of *TPS10* expression and hence stimulated the emission of two major TPS10 products [178]. Similarly, PpERF61 in peach (*Prunus persica*) could activate both *PpTPS1* and *PpTPS3* transcriptions simultaneously, leading to the accumulation of linalool during fruit ripening [179]. In addition to the ERF transcription factor, the expression of *PpbHLH1* in *Prunus persica* was observed to be significantly positively correlated with flavor-related linalool production, and PpbHLH1 could directly bind to the E-box (CACATG) in the *PpTPS3* promoter and activate its expression [42]. In tomato (*Solanum lycopersicum*), the downregulation of SlMYB75 can moderately increase the sesquiterpene accumulation (δ-elemene, β-caryophyllene, and α-humulene) through targeting the *SlTPS12*, *SlTPS31*, and *SlTPS35* genes [180]. SlSCL3, the SCL transcription factor, modulates the expression of terpene biosynthetic pathway genes via transcriptional activation and has similar expression patterns to those of *SlTPS12*, while neither direct protein–DNA binding nor interaction with known regulators has been observed [43]. More information about the biosynthesis of volatile terpenoids is presented in Table 1. However, it should be noted that the synthesis of volatile terpenoids is also associated with interactive transcription factor complexes and epigenetic modifications involving DNA methylation. It is interesting to further examine the multiple layers of transcriptional regulation for volatile terpenoids.

In *C. sinensis*, the miR171b-3p_2-DELLA-MYC2 and miR166d-5p_1-ABCG2-MYC2 modules were demonstrated to correlate with the terpenoid content, and these modules could enhance terpenoid biosynthesis in sun-withered leaves [184]. Xu et al. found that CsMYB13 and CsbHLH10 played possible crucial roles in the regulation of terpene metabolism based on metabolomic and transcriptome profiles [46]. Four CsMYBs, CsMYB193, CsMYB148, CsMYB147, and CsMYB68, showed high homology to the terpenoid regulator MYBs, and the synergistic functions of the *MYB*, *TPS*, and *MYC2* genes in terpenoid biosynthesis were confirmed based on the research of *Ectropis oblique* (EA) attacking tea leaves [47]. Based on transposase-accessible chromatin with sequencing (ATAC-seq) and DNA affinity purification sequencing (DAP-seq) analyses of the artificial hybrids of oolong tea, it was then speculated that MYB83, MYB58, MYB30, MYB81, ARF25, ARF27, and NAC45 might be the core transcription factors regulating the rate-limiting enzyme genes (*CsDXS*, *CsHMGS*, and *CsHMGR*) in terpenoid metabolic pathways [185]. More importantly, it was found that CsMYB83 contained a binding peak in the accessible chromatin region of *CsDXS*, which provided important evidence for studying its specific transcriptional regulation function [185]. Additionally, the metabolic phenotypes and gene expression profiles of tea leaves revealed a highly significant correlation between the expression of the NAC, ERF, WRKY, and bHLH transcription factors and key genes in the terpenoid biosynthesis pathway, suggesting the crucial roles of these transcription factors in aroma synthesis [48]. Although the comprehensive analysis of metabolomics and high-throughput sequencing technology has been used to identify many key transcription factors that might be involved in the biosynthesis of terpenes in tea plants, due to the lack of relevant evidence on the specific regulatory functions of these transcription factors, research on the transcription regulation of tea plant terpenes is still at the beginning stage. For example, it remains unknown whether these candidate transcription factors can directly bind to the structural genes in the terpene synthesis pathways, whether their transcriptional regulation functions are positive or negative, and whether there are interactive transcription factors involved in the regulatory network, and so on. Collectively, in-depth research on terpene biosynthesis in other horticultural crops has provided good examples for future investigations in finding answers to the above questions. Through conducting further in vivo and in vitro experiments on regulatory proteins, researchers can expect to extend and enrich the current understanding of the field.

## 5. Conclusions

In recent years, significant progress has been made in the study of terpene biochemistry in plants, which has promoted the transcriptional regulation of the terpene biosynthesis pathway in *C. sinensis*. Based on metabolomic and transcriptomic data, many terpene-related structural genes and transcriptional regulatory genes have been identified. The biotic and abiotic stress factors suffered by tea plants stimulate the production of volatile terpenes and have significant impacts on the quality of tea. Additionally, transcription factors including MYB, MYC, bHLH, NAC, ERF, and WRKY play important roles in the biosynthesis of tea plant terpenes. Despite the current knowledge of the close associations between the expression of individual or multiple genes involved in terpene biosynthesis, the transcriptional regulation research of tea terpene compounds remains limited. Overall, identifying additional transcription factors and structural genes would make it easier and more comprehensive to manipulate the transcription factors involved in pathway-level regulation. In the future, advanced biotechnologies and metabolic engineering technologies should be applied to explore the specific functions and binding sites of structural genes related to the MEP and MVA pathways.

## Figures and Tables

**Table 1:** Transcription factors regulating terpene synthesis-related genes in plants.

Transcription Factor	Accession Number	Target TPS Gene	Regulatory Activity	Compound	Plant	References
EREB58	GRMZM2G381441	*TPS10*	+	(*E*)-β-Farnesene; (*E*)-α-Bergamotene	*Zea mays*	[178]
PpERF61	Prupe.5G117800	*PpTPS1*; *PpTPS3*	+	Linalool	*Prunus persica*	[179]
PpbHLH1	Prupe.8G157500	*PpTPS3*	+	(*S*)-(+)-Linalool	*Prunus persica*	[42]
SlMYB75	FJ705320.1	*SlTPS12*; *SlTPS31*; *SlTPS35*	−	δ-Elemene; β-Caryophyllene; α-Humulene	*Solanum lycopersicum*	[180,181]
SlSCL3	Solyc12g099900	N.d	+	β-Ocimene; β-(*E*)-Caryophyllene; α-Humulene	*Solanum lycopersicum*	[43]
MYC2	At1g32640	*TPS11*; *TPS21*	+	(*E*)-β-Caryophyllene.	*Arabidopsis thaliana*	[39]
HY5	AT5G11260	*AtTPS03*	+	(*E*)-β-Ocimene	*Arabidopsis thaliana*	[41,182]
FhMYB21L1, FhMYB21L2	unigene-36442, unigene-49278	*FhTPS1*	+	Linalool	*Freesia hybrida*	[37]
AsMYC2	KP677282	*ASS1*; *TPS11*; *TPS21*	+	Volatile sesquiterpenes	*Aquilaria sinensis*	[183]
AaNAC2; AaNAC3; AaNAC4	KF319047; KF319048; KF319049	*AaTPS1*	+	Volatile terpene	*Actinidia chinensis*; *Actinidia arguta*	[40]

“+”, Transcription factor promotes the expression this target gene. “−”, Transcription factor has negative regulation activity. N.d., Not defined. TPS, Terpene synthase. ASS, Sesquiterpene synthase. TSB, Tryptophan synthase β-subunit.
